# Chemical proteomic identification of T-plastin as a novel host cell response factor in HCV infection

**DOI:** 10.1038/srep09773

**Published:** 2015-04-24

**Authors:** Young-Hwa Yoo, JiHyeon Yun, Chang No Yoon, Jun-Seok Lee

**Affiliations:** 1 Molecular Recognition Research Center, Korea Institute of Science and Technology, 39-1 Hawolgok-dong, Seoul, 136-791, Republic of Korea; 2 Department of Biological Chemistry, University of Science & Technology, 113 Gwahank-ro, Yuseong-gu, Daejeon 305-333, Republic of Korea

## Abstract

Hepatitis C virus (HCV) infection is the leading cause of chronic liver disease that currently affects at least 170 million people worldwide. Although significant efforts have been focused on discovering inhibitors of a viral polymerase (NS5B) or protease (NS3), strategies to cure HCV infection have been hampered by the limited therapeutic target proteins. Thus, discovery of a novel target remains a major challenge. Here, we report a method that combines transcriptome expression analysis with unbiased proteome reactivity profiling to identify novel host cell response factors in HCV infection. A chemical probe for non-directed proteomic profiling was selected based on genome-wide transcriptome expression analysis after HCV infection, which revealed noticeable alterations related to disulfide bond metabolism. On the basis of this result, we screened the proteome reactivity using chemical probes containing thiol-reactive functional groups and discovered a unique labeling profile in HCV-infected cells. A subsequent quantitative chemical proteomic mapping study led to the identification of a target protein, T-plastin (PLST), and its regulation of HCV replication. Our approach demonstrates both a straightforward strategy for selecting chemical probes to discriminate disease states using a model system and its application for proteome reactivity profiling for novel biomarker discovery.

Hepatitis C virus (HCV) infection is the leading cause of liver transplantation in the United States, and almost 80% of patients suffer a persistent chronic infection that results in fibrosis, cirrhosis, and hepatocellular carcinoma.^1^ The currently available treatments use a combination of an HCV protease inhibitor with ribavirin and PEGylated alpha interferon to disrupt virus replication, but the therapy is effective in only half of the people infected with HCV genotype 1, and even in those patients the efficacy is limited.^2^ Two recently approved drugs targeting the HCV protease (telaprevir and boceprevir) showed considerably improved curative effects,^3^,^4^,^5^ however, there are still unmet needs for more effective antivirals. Despite intensive efforts over the last decades, strategies to cure HCV infection have been impeded due to the lack of a detailed understanding of the biology of the HCV infection process. Most previous attempts were focused on discoveries of inhibitors of viral polymerases or proteases because of the narrow scope of known therapeutic targets.^6^,^7^,^8^ Alternative targets are host cell factors that play roles in HCV replication. HCV is a positive-strand RNA virus of the *Flaviviridae* family that contains 9.6 kb of RNA.^9^ HCV encodes a single polypeptide protein that is subsequently cleaved into structural (core, E1, and E2) and nonstructural (NS2, NS3, NS4A/B, and NS5A/B) subunits by both viral and host proteases.^10^ Briefly, viral enzymes (NS2/NS3 and NS3 protease) cleave the nonstructural proteins from the polypeptide protein to generate mature forms, whereas host cell enzymes are responsible for processing structural proteins.^11^,^12^ Thus, host cell factors are closely involved in HCV replication, and they have high potential as new therapeutic targets for regulating HCV infection.

To examine host cell responses to HCV infection, biologists have utilized conventional high throughput (HTS) techniques, such as gene or proteomic expression profiling.^13^,^14^,^15^,^16^,^17^ These approaches have unveiled many important host-HCV interactions,^18^,^19^ but these techniques provide only the perturbations in expression abundance despite the fact that the HCV replication process is highly regulated by various post-translational modifications (PTM) and proteolysis. To directly monitor the catalytic activities of enzymes, an activity-based protein profiling (ABPP) method was applied to the protease and fatty acid synthase superfamily;^20^,^21^ this analysis revealed the differential activity of those enzymes together with several small-molecule regulators.^22^,^23^ Although ABPP can provide unique insight into the intact metabolic status during HCV infection, this approach still has drawbacks. First, target enzymes of ABPP probes are limited to only a few enzyme superclasses at the moment.^24^,^25^ Second, the pathological features of many diseases, such as HCV infection, are not well characterized, which makes it difficult to choose proper chemical probes.

As a complementary method for enzyme activity profiling, undirected proteomic profiling has unique merits in terms of the diversity of target proteins. It has been reported that proteome reactivity can be monitored using various small-molecule electrophiles,^26^,^27^,^28^ and the usefulness of identifying functional cysteine residues^29^ or discriminating pathogens has been demonstrated.^30^ In particular, we found that distinct pathological samples produced fingerprint signatures of proteome reactivity patterns.^30^ Currently, the major bottleneck step of undirected profiling for disease models is identification of proper electrophiles to maximize the discriminant signature. We envisioned that conventional HTS data could provide insights for selecting desirable chemical probes. Here, we demonstrated a strategy that combines transcriptome expression assisted non-directed proteomic profiling (TEAnDPP) to identify host cell response factors in genotype 2a HCV infection (Figure 1).

To determine small-molecule electrophiles, we initiated our studies by exploring the transcriptome analysis of the human hepatoma cell line (Huh7.5) expressing the HCV2a subgenomic replicon (APC140 cells: Huh7.5 cells containing a genotype 2a subgenomic replicon in bicistronic configuration; HuhHuh7.5/J6/JFHEMCVIRESRlucNeo). The replicon system was developed for stable expression of HCV2a proteins in host cells,^31^ and we chose this system for the ease of culture and for the maintenance of homogeneity in the viral protein expression. Total RNA was extracted from control Huh7.5 cells and Huh7.5 cells expressing the HCV2a replicon (APC140 cells), and the genome-wide transcriptome expression levels were measured using an Illumina Human HT12 expression bead array (data are freely available in an NCBI GEO repository: GSE62546). Based on the statistical significance and the fold change values of the expression levels, we identified 541 differentially expressed genes (DEGs) out of 47,000 total genes with high reproducibility from duplicated experiments (Fig S1a-b). Rather than focusing on the strongly responsive genes, we investigated the biological functions of all 541 DEGs using DAVID gene enrichment analysis to determine the general responses of the host cell.^32^,^33^ DAVID is a bioinformatics tool for integrative functional analysis of a large gene list. Gene ontology analysis revealed that biological pathways related to cellular hormone metabolism and chromatin assembly were considerably perturbed (Fig S1c-d). Furthermore, the functional category of the most significantly enriched DEG cluster was disulfide bond processing (83 genes in 541 DEGs; Table 1). Because gene enrichment analysis showed remarkable distinctions in cellular thiol metabolism, we hypothesized that thiol-reactive probes could generate differential proteome reactivity signatures upon HCV infection. Therefore, we chose α−iodoacetamide (IA), vinyl sulfone (VS), and benzyl halide (BH) functional groups that selectively label free thiol groups.

Unlike other diseases that cause dramatic pathological changes, HCV infection tends to induce subtle and chronic interference in the host cell. In our cell line model, the HCV replicon expression did not produce noticeable changes in the cell morphology or the total proteome band pattern, which was measured using coomassie staining (Fig 2a). To visualize the non-directed proteome reactivity fingerprints in both cell lines, we used two oppositely charged fluorophores for individual IA, VS, and BH functional groups (Figure S2). We administered each probe at a concentration of 1 μM for 30 min in live Huh7.5 and APC140 cells, and the cell lysates were separated using SDS-PAGE. Proteome reactivity signatures were obtained using fluorescence gel imaging with the proper excitation and emission filters (Fig 2b-d). All three electrophiles generated unique proteome reactivity patterns for control Huh7.5 cells (Fig 2b-d: left lanes). In general, the VS groups exhibited the most intense and numerous bands among the three motifs due to their intrinsic high electrophilicity, and IA showed faint bands and the least number of labeled bands. Notably, the undirected protein targets that were labeled by probes significantly differed depending on the charge state of the fluorophores. Our particular interest was the relative proteome reactivity changes between Huh7.5 cells with and without expression of the HCV2a replicon, and all 6 thiol-reactive probes generated distinct labeling patterns, as we anticipated. This observation was also supporting the finding from the transcriptome analysis that showed that the reactivities of many cellular thiols were altered by thiol metabolism upon HCV replication.

To investigate the host cell factors that are selectively up-regulated upon HCV replication, we employed competition-based quantitative chemical proteomic profiling geared to determine the identity of labeled proteins (Figure S3). Inspired by the competitive isoTOP-ABPP strategy,^34^ we adapted the protocol utilizing stable-isotope labeling of amino acids in cell culture (SILAC). SILAC involves differential labeling of proteins with stable isotopes of different mass to generate isotopically “heavy” and “light” samples. Because the Flu-IA probe exhibited the most prominent change upon HCV2a replicon expression, we then tried to identify the protein targets of the Flu-IA probe (Fig 2-b). Control Huh7.5 cells were grown in medium containing “heavy” isotopes of arginine (^13^ C_6_,^15^N_4_) and lysine (^13^C_6_), and APC140 cells were grown in “light” media. As illustrated in Figure S3, we conducted two-way competition experiments in both Huh7.5 cells and APC140 cells. Flu-IA was administered to live cells: either “light” isotope-labeled APC140 cells or “heavy” isotope-labeled Huh7.5 cells at a 1 μM concentration for 30 min, and whole-cell lysates were subsequently incubated with an excess amount of biotin polyethylene oxide IA (Biotin-IA, 100 μM) to enrich proteins that could form a covalent bond with the IA functional group but were not labeled with Flu-IA. Then, cells that were not treated with Flu-IA were prepared as a control, and the lysates were also labeled with excess Biotin-IA to enrich proteins that could form a covalent bond with the IA functional group, which included Flu-IA targets in this case. The same quantities of proteins were mixed, and enriched biotinylated proteins were separated by affinity purification using avidin-coated agarose beads. Following on-bead trypsin digestion, the peptide mixtures of enriched proteins were separated by nano-flow HPLC and analyzed with using an Orbitrap mass spectrometer.

From the SILAC-based quantitation results, proteins exhibiting competition in both cases were considered to be non-directed target proteins of Flu-IA. In all, 71 proteins were identified from the competition experiment in “light” APC140 cells having a SILAC ratio (Heavy/Light) greater than two folds (Table S2a), and 46 proteins were discovered from the competition in “heavy” Huh7.5 cells having a SILAC ratio (H/L) lower than 0.5 (Table S2b). Both competition experiments were performed in triplicate, and the proteins identified in both cases were 26 proteins with sizes ranging from 18.5 kDa to 273.3 kDa (Table S2-c), including previously reported host cell factors for HCV infection, such as chloride channel protein 1,^23^ fatty acid synthase,^21^,^35^ heat-shock protein 90,^36^ protein disulfide-isomerase,^20^ and thioredoxin peroxidase.^37^ From these proteins, we were especially interested in the one that showed a strong fluorescence band in an SDS gel (Fig 2-b). The protein size of the marked band in Figure 3-b was approximately 70 kDa, and there was one protein in that range, plastin-3 (i.e., T-plastin). We further confirmed the identity of the corresponding band by western blot (Fig S4), but we could not find the exact modification site, possibly due to the low ionization efficiency of the charged modification.

The plastin family comprises actin-bundling proteins that are critical to actin regulation in eukaryotes.^38^ Plastins are evolutionary conserved and expressed throughout eukaryotes; thus, plastins have been considered one of the key regulators that have a fundamental cellular function, but functional studies of plastins are still at an early stage.^39^,^40^ Plastins consist of N-terminal EF-hand Ca^2+^-binding domains and actin-binding domains (ABD).^41^ Unlike other ABD-containing proteins, plastins contain two tandem repeats of ABD, which are involved in cross-linking actin filaments into bundles.^42^

Because HCV NS3 and NS5A proteins interact with microtubules and actin filaments to transfer the replication complex to various subcellular regions,^43^ we postulated that up-regulating of T-plastin might have a cooperative influence on HCV replication. To validate the collaborative effect of T-plastin, we examined the dependence of HCV replication efficiency on perturbations of intact T-plastin. An RNAi knock-down experiment of T-plastin resulted in greater than 50 % inhibition of the HCV replication efficiency, as indicated by the *Renilla* luciferase activity encoded in the HCV2a replicon (Fig 3-a). The HCV replication efficiency was also altered by the Flu-IA modification of T-plastin, which might induce conformational changes similar to those of other endogenous PTMs that disturbed the actin-bundling activity (Fig 3-c).^40^ In addition, it was previously reported that an actin polymerization inhibitor, cytochalasin D, caused dose-dependent inhibition of the HCV replication efficiency at micromolar concentrations.^44^ Taken together, these observations suggested that the actin-bundling effect of T-plastin facilitated the HCV replication process, and selective perturbation of T-plastin could be an alternative strategy to treat HCV infection.

In summary, we have demonstrated a robust strategy that combines transcriptome expression signature analysis and non-directed proteome reactivity profiling to discover a novel host cell response marker for HCV infection. Based on the unique signature of thiol metabolism, we chose cross-reactive thiol-targeting probes to obtain a proteome reactivity profile, and we discovered T-plastin as a novel host cell response factor. Interfering with the expression abundance or exogenous modification of T-plastin attenuates HCV replication, which suggests that modulating this protein may provide a strategy for treating HCV infection. We are currently working on discovering small-molecule ligands that target T-plastin and on applying TEAnDPP to diverse infectious disease models.

## Author Contributions

**Figure 1:**
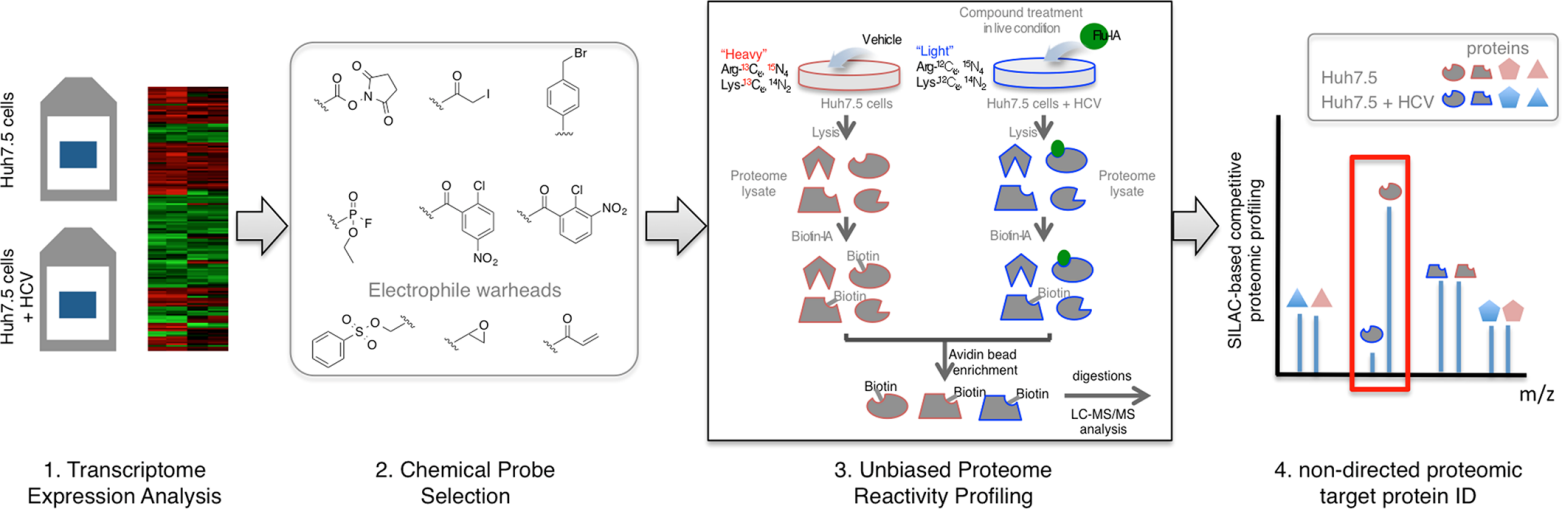
Schematic of the transcriptome expression assisted non-directed proteomic profiling (TEAnDPP) strategy for identifying host cell response factors.

**Figure 2:**
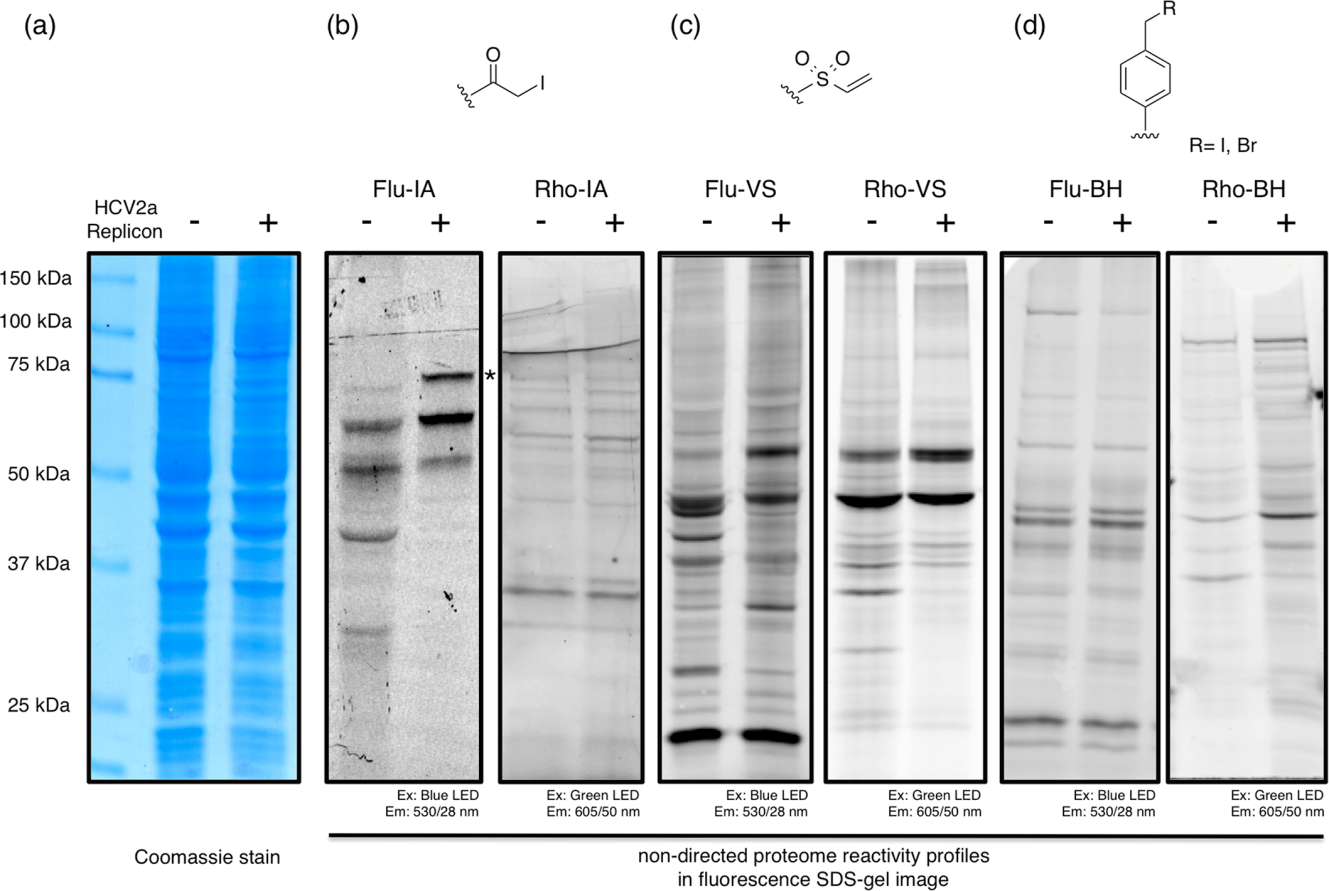
Investigation of the non-directed proteome reactivity to iodoacetamide (IA), vinyl sulfone (VS), and benzyl halide (BH) functional groups. (a) Coomassie staining of Huh7.5 cells without (left) and with expression of the HCV2a replicon (right). (b-d) In-gel fluorescence image of the probe labeling in Huh7.5 cells without (left) and with expression of the HCV2a replicon (right).

**Figure 3:**
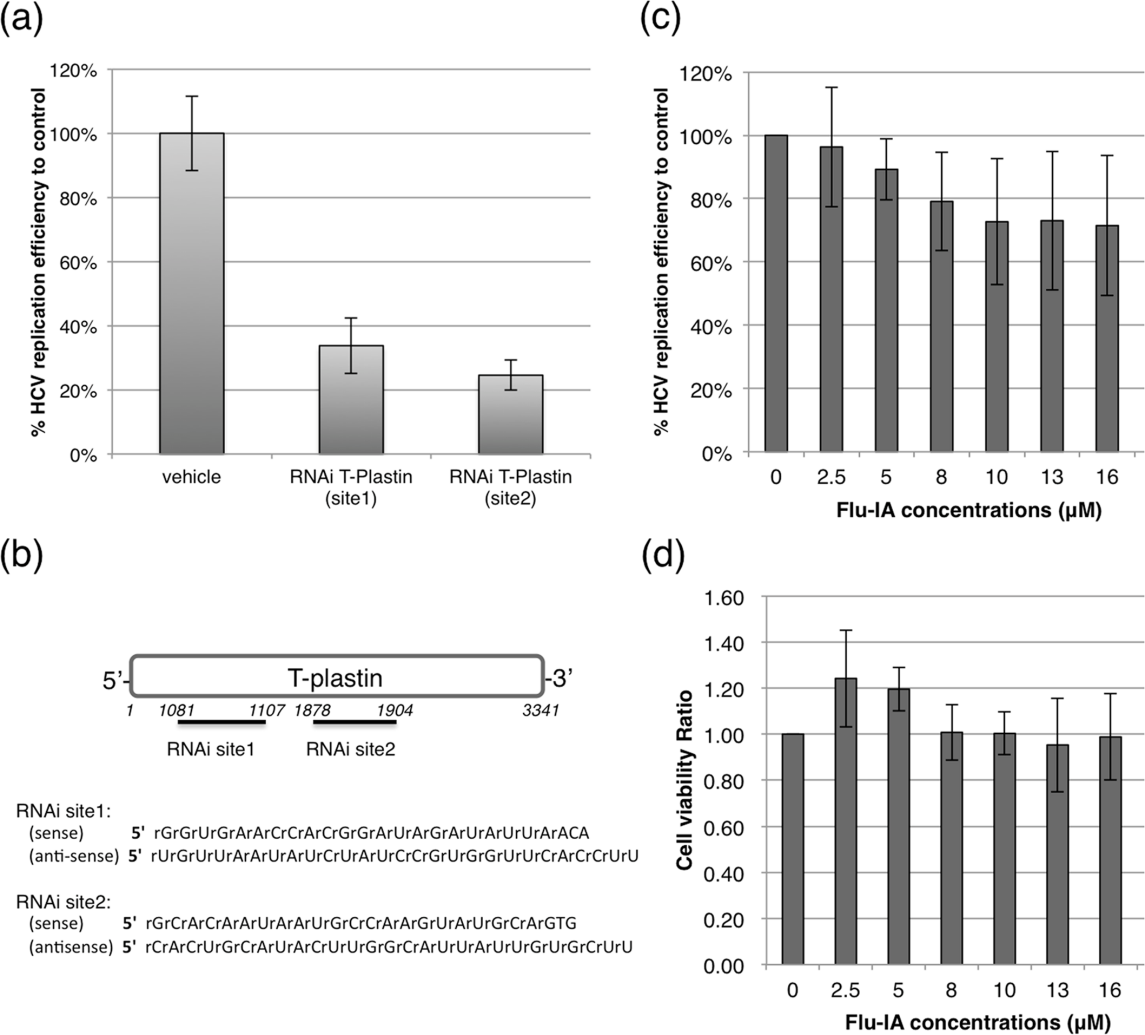
Inhibition of HCV replication. (a) Effect of T-plastin knock-down measured by *Renilla* luciferase activity. (b) Two target sites of T-plastin RNAi. (c) Dose-dependent HCV replication inhibition effect of prolonged Flu-IA treatments. (d) Cell viability test by an MTT assay in response to serial concentrations of Flu-IA treatment. All mean and standard deviation data were obtained from quadruplicate experiments (*N* = 4).

J.-S.L. designed the TEAnDPP workflow and conducted the entire proteomic profiling study with Y.-H.Y. C.N.Y. and J.-S.L. contributed reagent/material/analysis tools. Y.-H.Y. and J.-S.L. wrote the main manuscript text, and J.Y. and Y.-H.Y. prepared figures 1-3. All authors reviewed the manuscript.

## Additional information

**Accession codes:** Transcriptome expression data are freely available in the NCBI GEO repository (GSE62546). All protein lists and quantitative analysis results are also available in the SI.

## Supplementary Material

Supplementary Information Supplementary Info

Supplementary Information Supplementary Dataset I

Supplementary Information Supplementary Dataset II

## Figures and Tables

**Table 1:** DAVID gene enrichment scores of functional category keywords. In total, 541 DEGs were analyzed against Uniprot functional categories and enriched gene lists were generated for each functional category.

Functional Categories (Uniprot)	Enrichment Score of Cluster	Gene Count	Genes
SP_PIR_KEYWORDS: disulfide bond	3.810	83	A2M, MICB, NRTN, GABRB1, EDN1, JAG1, DLK1, CXCL10, SLC7A7, UNC5B, GSN, HAMP, CNTNAP2, PLA1A, CYGB, LOXL4, FABP5L2, CFD, CEACAM1, KNG1, MATN3, STC2, ICAM2, LYZ, OLFML2A, TNFRSF14, HEPACAM2, HLA-E, SIRPA, MMP11, AADAC, INHBE, IGF2R, ULBP1, LRP11, ULBP2, TFPI, ROR1, VCAN, PRNP, CTSH, LUM, KITLG, CXCL6, LEAP2, AHSG, COL9A2, NPTX2, FGB, TFF2, TFF3, THBS1, ANGPTL2, GCNT1, CD7, ANGPTL4, HPN, GLRB, LGALS3, EFEMP1, CELSR2, FZD2, C4BPA, COL4A6, FZD7, COL4A5, NOTCH3, DNASE2, DKK1, COL14A1, GPR37, PTP4A3, PI3, LASS1, C1RL, EPOR, ADM2, GDF15, FABP5, IGFBP4, PON3, CD14, HABP2, VLDLR
SP_PIR_KEYWORDS: hydroxylation	2.521	8	KNG1, COL9A2, COL14A1, COL1A2, CELSR2, COL2A1, COL4A6, COL4A5
SP_PIR_KEYWORDS: methylation	2.339	18	FUS, HIST1H2AC, HIST2H2AA3, HIST2H2AA4, HIST1H2BD, HIST1H1C, EEF1A2, RHOQ, HIST2H4A, RPL29, RND2, HIST1H2BK, TAF15, HIST2H2BE, PPP2CA, HIST2H2AC, LOC399942, THOC4, RASD1, HIST1H4H
SP_PIR_KEYWORDS: lyase	2.261	11	DDC, CTH, ENO2, ACMSD, HAL, GUCY1A3, ENO3, CA2, GUCY2C, PCK2, GAD1
SP_PIR_KEYWORDS: microsome	1.912	8	AADAC, UGT2B17, CYP1A1, UGT2B11, HSD17B6, CYP26A1, UGT2B4, UGT2B7
