# Supplementary material for: Chemical proteomic identification of T-plastin as a novel host cell response factor in HCV infection
Source: Sci Rep. 2015 Apr 24;5:9773. doi: 10.1038/srep09773 (PMC4408979; doi:10.1038/srep09773)

Supporting Information:

Chemical proteomic identification of T-plastin as novel host cell response factor in HCV infection

Young-Hwa Yoo[†,§], JiHyeon Yun[†,§], Chang No Yoon[†], Jun-Seok Lee[†, ‡]*

[†]Molecular Recognition Research Center, Korea Institute of Science and Technology, 39-1 Hawolgok-dong, Seoul, 136-791, Republic of Korea
[‡]Department of Biological Science, University of Science & Technology, 113 Gwahank-ro, Yuseong-gu, Daejeon 305-333, Republic of Korea

Email: junseoklee@kist.re.kr

1. **Materials and general procedures**

2. **Figure S1.** Transcriptome expression analysis between Huh7.5 cells and Huh7.5 cells expressing HCV2a replicon.

3. **Figure S2.** Structures of chemical probes for proteome reactivity profiling.

4. **Figure S3.** SILAC-based competitive chemical proteomic method for non-directed target protein ID

5. **Figure S4.** Target protein validation for T-Plastin.
   (a,b,c) Western blot analysis
   (d) SILAC quantitaion of T-Plastin in competition experiment on "heavy" Huh7.5
   (e) SILAC quantitaion of T-Plastin in competition experiment on "light" APC140

6. **Table S1.** Fold change values of transcriptome expression between Huh7.5 cells and Huh7.5 cells expressing HCV2a replicon.

7. **Table S2-(a).** SILAC-based quantitation result of proteins showing H/L > 2 upon Flu-IA treatment in "light" Huh7.5 expressing HCV2a replicon

8. **Table S2-(b).** SILAC-based quantitation result of proteins showing H/L > 1/2 upon Flu-IA treatment in "heavy" Huh7.5 control cell.

9. **Table S2-(c).** Protein list targeted by Flu-IA

Materials and general procedures

1. Materials

Unless otherwise noted, all chemicals and solvents were obtained from commercial suppliers (Sigma-Aldrich Inc) and used without further purification. All fluorophore probes were purchased from SigmaAldrich Inc., Invitrogen Inc., and Bioacts Inc. Details product informations are here: Fluorescein-iodoacetamide (Flu-IA; Sigma catalogue #: I30451), tetramethyl rhodamine-iodoacetamide (Rho-IA; Sigma catalogue #: T6006), (4 or 5)(2-(vinylsulfonyl)ethyl)carbamoyl-fluorescein (Flu-VS; Bioacts Inc. catalogue #: FPG456), and (4 or 5)(2-(vinylsulfonyl)ethyl)carbamoyl-tetraethylrhodamine (Rho-VS; Bioacts Inc. catalogue #: FPR560), 5-(bromomethyl) fluorecein (Flu-BH; Invitrogen catalogue #:B1355), mitotracker CMXRos (Rho-BH; Invitrogen catalogue #: M7512). Biotin polyethyleneoxide iodoacetamide (Biotin-IA) was purchased from Sigma-Aldrich (Catalogue #: B2059).

2. Cell culture and non-directed proteome reactivity profiling

Huh7.5 cells were cultured in growth medium (DMEM supplemented with 10 % fetal bovine serum and 100 IU/ml penicillin). APC140 cells (Huh7.5 cells stably expressing HCV2a replicon J6/JFH-1 strain; Huh7.5/J6/JFHEMCVIRESRlucNeo) were grown in growth medium (DMEM supplemented with 10 % fetal bovine serum, 100 IU penicillin, 0.5 g/L Geneticin). Both cells were maintained in a 5 % CO_2 at 37 °C, and sub-cultured three times per week. Particularly, APC140 cells were maintained maximum confluency of 80 % as the Apath protocol (Protocol #: CC1002). The Huh7.5 expressing HCV2a replicon cell line (APC140 cell line) was kindly provided by Dr. Charles Rice and Dr. Takaji Wakita via Apath, LLC.

For non-directed proteome reactivity profiling, cells were incubated with 1 μM fluorescence probes (Flu-VS, Flu-IA, Flu-BH, Rho-VS, Rho-IA or Rho-BH) for 30 min. Next, unreacted compounds were washed out using PBS twice. Then, cells were lysed by pulsed ultra-sonication for 1 min in 10 mM PBS buffer containing 1x compete protease inhibitors cocktail (Sigma-Aldrich: P2714). After ultra-sonication, membranes and debris were removed by centrifugation (18,000 g for 20 min at 4 °C), and soluble fraction of proteome was obtained. Protein concentration of lysate was quantified using Nanodrop™ Lite. After quantifying protein concentration, lysates were mixed with the Laemmli buffer (62.5 mM Tris-HCl, pH 6.8, 20% glycerol, 2% SDS, 2 mM DTT, Halt phosphatase inhibitor and Roche proteinase inhibitor cocktail), and boiled at 98 °C for 2 min. An equal amount of protein was loaded on 12 % gel and separated by SDS-PAGE. Fluorescence signals were obtained using Chemi-Doc (BioRad, Inc.) using proper excitation LED (Flu: Blue LED, Rho: Green LED) and emission filter (Flu: 530/28 filter, Rho: 605/50 filter).

3. RNA preparation for transcriptome expression analysis

Total RNA was extracted using Trizol (Invitrogen Life Technologies, Carlsbad, USA), purified using RNeasy columns (Qiagen, Valencia, USA) according to the manufacturers' protocol. After processing with DNase digestion, clean-up procedures, RNA samples were quantified, aliquot and stored at -80°C until use. The obtained total RNA was submitted to Macrogen Inc. (Seoul, South Korea) for transcriptome expression analysis using Illumina Human HT12 expression v.4 bead array (Illumina, Inc., San Diego, USA).

4. Microarray raw data preparation and Statistic analysis

Raw data were extracted using the software provided by the manufacturer (Illumina GenomeStudio v2011.1 (Gene Expression Module v1.9.0)). Probes signal value was transformed by logarithm and normalized by quantile method. Statistical significance of the expression data was determined using 1-way ANOVA test and fold change in which the null hypothesis was that no difference exists among groups. False discovery rate (FDR) was controlled by adjusting p value using Benjamini-Hochberg algorithm. The 1-way anova test, tukey HSD test were applied. K-means clustering analysis was performed using MacQueen algorithm to compare the expression profile among groups. Gene-Enrichment and Functional Annotation analysis for significant probe list was performed using DAVID (http://david.abcc.ncifcrf.gov/) database. All data analysis and visualization of differentially expressed genes was conducted using R 2.15.3 (www.r-project.org), Bioconductor modules.

5. Competitive chemical proteomic mapping for non-directed target proteins of Flu-IA

5-1. Competition experiment in APC140 cells

(a) Peptide digests sample preparation

For SILAC experiment, Huh7.5 cells were cultured in growth medium substituted all Arg ($^{13}C_6$, $^{15}N_4$) and Lys ($^{13}C_6$) for "heavy" isotope for three weeks prior to experiment (DMEM (Thermo, 88420) supplemented with Lys ($^{13}C_6$; Thermo, 89988), Arg ($^{13}C_6{}^{15}N_4$; Thermo, 89990), 10 % dialyzed fetal bovine serum (Gibco, 26400044) and 100 IU/ml penicillin). APC140 cells were grown in growth medium labeled with "light" isotope of Lys and Arg for three weeks (DMEM (Thermo, 88420) supplemented with Lys ($^{12}C_6$; Thermo, 89987), Arg ($^{12}C_6{}^{14}N_4$; Thermo, 89989), 10 % dialyzed fetal bovine serum (Gibco, 26400044), 100 IU penicillin, 0.5 g/L Geneticin). Both cells were maintained in a 5 % CO_2 at 37 °C, and sub-cultured three times per week.

Flu-IA compound was incubated in live APC140 cells at 1 uM concentration for 30 min. Then, cells were lysed by pulsed ultra-sonication for 1 min in 10 mM PBS buffer containing 1x compete protease inhibitors cocktail (Sigma-Aldrich: P2714). After ultra-sonication, membranes and debris were removed by centrifugation (18,000 g for 20 min at 4 °C), and soluble fraction of proteome was obtained. The supernatant were transferred to fresh e-tube, and proteome lysate concentration was quantified using Nanodrop™ Lite. Control Huh7.5 cells were lysed without compound treatment using same protocol as APC140 cells.

After protein concentration was determined for the sample, non-directed protein targets were labelled by excess amount of biotin-IA. To soluble proteome (1 mg/ml) of Huh7.5 and APC140 cells, biotin-IA probes were added to final concentration of 100 mM and incubated in room temperature for 30 min. After reaction was finished, unreacted biotin-IA was removed using size exclusion membrane filter, 5K-Centricon (Millipore Inc.). After removal of unreacted biotin-IA, identical amount of proteome was mixed in separate e-tube. Then, the mixed proteome were suspended with avidin agarose beads, and further incubated 1 hour at room temperature. Non-specific binding proteins were washed out using washing buffer A, B, C (washing buffer A: 1% SDS 1ml x3; washing buffer B: 6M Urea 1ml x3; washing buffer C: PBS 1ml x5). Then, avidin beads were transferred to low adhesion e-tube, and suspend the beads in 500 ul 6M Urea in PBS. Bead bound proteins were reduced by adding 500 mM DTT 10 ul, and incubated for 15 min at 65 °C. Next, the bead bound proteins were alkylated by add 750 mM iodoacetamide 16 ul, and incubated for 30 min at room temperature under dark. Discard supernatants, and re-suspend the beads using 100 mM ammonium bicarbonate buffer. Finally, bead bound proteins were digested by mass-grade trypsin (5 ug; Catalogue #: V5280, Promega Inc.) for overnight at 37 °C. After overnight digestion, formic acid was added to the sample to 5 % final concentration. The tryptic digests were desalted using Ziptip C18, and stored at – 80 °C until mass spectrometry analysis.

(b) Liquid chromatography-tandem mass spectrometry (LC-MS/MS) analysis

LC-MS analysis was performed on a nanoAcquity (Waters, inc) LC system coupled with Q-exactive Orbitrap mass spectrometer (ThermoScientific Inc.). Proteome disgests were loaded onto a fused silica desalting column packed with 2 cm C18 reverse phase resin (Phenomenex Inc.), and peptides were separated on a reverse phase column (100 μm fused silica packed with 15 cm C18 resin). Peptide separation condition was a gradient 5-50% Buffer B in Buffer A (Buffer A: water, 0.1 % formic acid; Buffer B: acetonitrile, 0.1% formic acid), and the flow rate through the column was set to 250 nl min-1, and spray voltage was set to 2.2 kV. Neither sheath gas nor makeup liquid was used. MS data acquisition began 0.1 min after the sample was injected and continued throughout analysis for data dependent analysis (DDA) mode. DDA parameter was set for each full MS scan (350 – 1800 MW) followed by 10 MS/MS fragmentation scans for most intense ions with dynamic exclusion enabled for 30 sec. The resulting MS/MS data was searched using the in-house SEQUEST™ server using IPIHuman proteome sequence database (IPIHuman v3.87) using Proteome Discoverer software (v.1.4.0.288). A maximum of two missed cleavages were allowed and fully tryptic peptides were considered with dynamic modification of acetyl at N-terminal. The maximal mass tolerance in MS mode was set to 10 ppm, and fragment MS/MS tolerance was set to 0.36 Da for HCD data. For percolator parameters, the maximum delta Cn value was set as 0.05 and false discovery rates (FDR) were specified as 0.01. SILAC quantification was performed using Precursor Ion Quantifier module in Proteome Discoverer. Based on "Heavy/Light" quantitation ratio, proteins showing SILAC ratio (H/L) > 2 from all triplicated experiments were considered as potential competitive target protein of Flu-IA. Overall protein lists are available in Table S2-a.

5-2. Competition experiment in Huh7.5 cells

All experimental protocols are identical; except Flu-IA was treated to "heavy" labeled Huh7.5 cells. And proteins showing SILAC ratio (H/L) < 1/2 from all triplicated experiments were considered as potential competitive target protein of Flu-IA in Huh7.5 cells. Overall protein lists are available in Table S2-b.

5-3. Determination of Flu-IA target proteins

Proteins that exhibited SILAC ratio (H/L) > 2 from APC140 (L) competition experiment and SILAC ratio (H/L) < 1/2 in Huh7.5(H) competition experiment were considered as non-directed target protein of Flu-IA. Total 26 proteins were identified, and listed in Table S2-c.

6. Western blot analysis for T-plastin

Huh7.5 and APC140 cells were lysed by pulsed ultra-sonication for 1 min in 10 mM PBS buffer containing 1x compete protease inhibitors cocktail. After quantifying protein concentration, lysates were mixed with the Laemmli buffer (62.5 mM Tris-HCl, pH 6.8, 20% glycerol, 2% SDS, 2 mM DTT, Halt phosphatase inhibitor and Roche proteinase inhibitor cocktail), and boiled at 98 °C for 2 min. An equal amount of protein was loaded on 12 % gel and separated by SDS-PAGE. Next, we carried out western blotting analysis using corresponding primary antibody and secondary HRP antibody. T-plastin was visualized using anti T-plastin antibody (Abcam, ab45769), and beta-actin, loading control, was visualized using anti beta-actin (Abcam, ab25894).

7. *Renilla* luciferase assay

About 80 % confluent cells in 6 well plates were used for *Renilla* luciferase assay. For RNAi experiment, cells were transfected with two kinds of RNAi reagents targeting distinct binding site of T-plastin transcript using Lipofectamine 2000 (Invitrogen, 11668027). Two RNAi sequences were designed by IDT RNAi Design Tool (https://www.idtdna.com/Scitools/Applications/RNAi/). RNAi site1: (sense) rGrGrUrGrArArCrCrArCrGrGrArUrArGrArUrArUrUrArACA (antisense) rUrGrUrUrAr ArUrArUrCrUrArUrCrCrGrUrGrGrUrUrCrArCrCrUrU, RNAi site 2: (sense) rGrCrArCrArArUrArA rUrGrCrCrArArGrUrArUrGrCrArGTG (antisense) rCrArCrUrGrCrArUrArCrUrUrGrGrCrArUrUrA rUrUrGrUrGrCrUrU. For Flu-IA treatment experiment, serial concentrations of Flu-IA were treated to 80 % confluent cells in 6 well plates for 2 hours prior to lysis, and measured luciferase activity.

Renilla luciferase assay was carried out by following the protocols provided by manufacturer (Promega, E2820). Briefly, cells were lysed using *Renilla* Luciferase Assay Lysis Buffer supplemented with 1x compete protease inhibitors cocktail. After protein quantification, an equal amount of proteins in 25 ul lysates were transferred to white flat bottom 96 well plates, and add 100 ul of 1x *Renilla* Luciferase Substance Buffer right before measuring chemi-luminescence signal. Chemi-luminescence signals were obtained by FlexStation microplate reader (Molecular Device, Inc).

8. Cell viability assay

Cytotoxicity effects of Flu-IA compound were measured using Non-Radioactive Cell Proliferation Assay kit (Promega, G4000). To measure cell toxicity of Flu-IA compounds, serial concentrations of Flu-IA were incubated in 80 % confluent cells in 6 well plates for 2 hours. Then, colorimetric tetrazolium reagent was added to media and incubated for 2 hours 37 °C, 5% CO_2 condition. Next, color development was terminated by subsequent addition of Stop Solution. Colorimetric changes were measured at 595 nm absorbance values by FlexStation microplate reader (Molecular Device, Inc).

Figure S1. Transcriptome expression analysis between Huh7.5 cells and Huh7.5 cells expressing HCV2a replicon. (a) Differentially expressed genes upon HCV replicon expression. (b) Heatmap and hierarchical clustering results of DEGs (541 genes selected by the criteria of "fold change ≥ 2" and "p-value < 0.05"). Hierarchical clustering was performed using Euclidian distance of fold change values. (c) Bar graph of gene-enrichment analysis for gene ontology terms (http://www.geneontology.org) in the 541 DEGs. (d) Enriched gene lists for each categories of GO term.

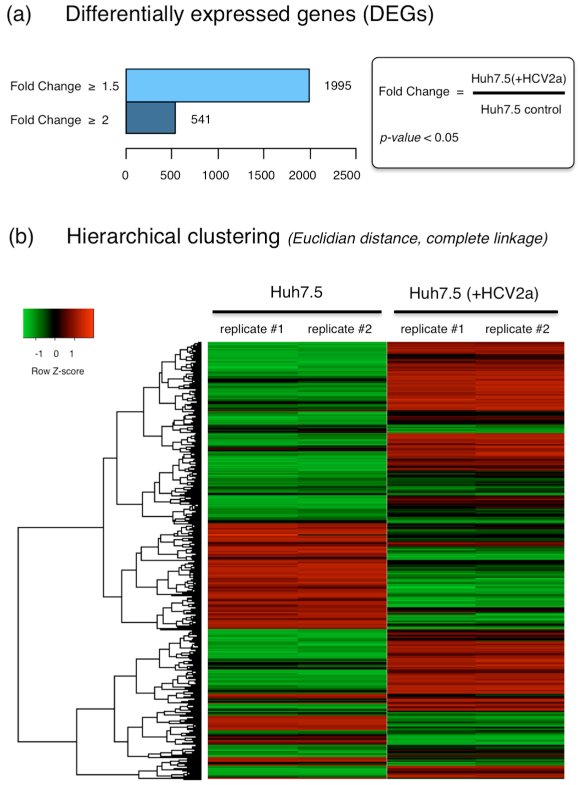

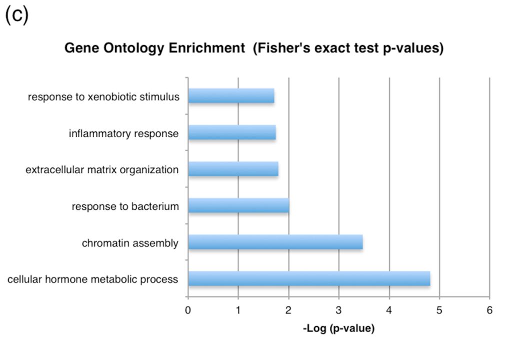

(d)

GO Term (GOTERM_BP_FAT)	-Log(P-value)	Genes
cellular hormone metabolic process	4.815425449	SCPEP1, APOA1, AKR1C4, CYP1A1, RBP1, UGT2B11, HSD17B6, CYP26A1, UGT2B4, COMT
chromatin assembly	3.473025489	H1F0, HIST1H2AC, HIST2H2AA3, HIST2H2AA4, HIST1H2BD, HIST1H1C, HIST1H2BK, HIST2H2BE, HIST2H2AC, H2AFX, HIST2H4A, HIST1H4H
response to bacterium	2.006066561	PLD1, CYP1A1, HIST1H2BK, HIST2H2BE, HAMP, SNCA, PLCG2, LYZ, COMT, LEAP2, CD14, VLDLR
extracellular matrix organization	1.793919693	COL14A1, LGALS3, LUM, COL1A2, OLFML2A, COL2A1, LOX, COL4A6
inflammatory response	1.744067742	KNG1, A2M, LYZ, SAA4, C4BPA, CXCL6, GAL, AHSG, CXCL10, NUPR1, C1RL, CFD, THBS1, IGFBP4, CD14, SPP1
response to xenobiotic stimulus	1.713329863	CYP1A1, UGT2B11, UGT2B4, NQO1

Figure S2. Structures of chemical probes for proteome reactivity profiling

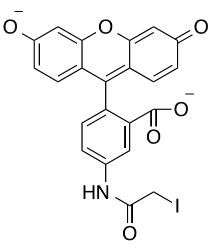

Flu-IA

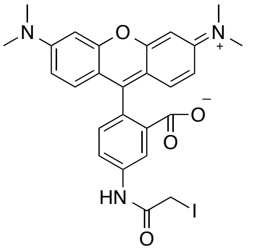

Rho-IA

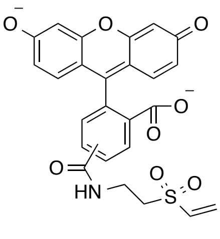

Flu-VS

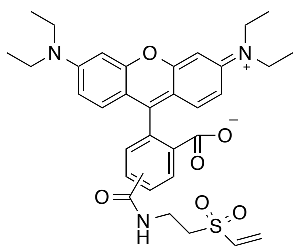

Rho-VS

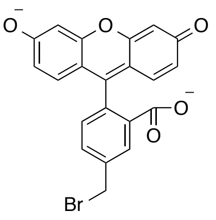

Flu-BH

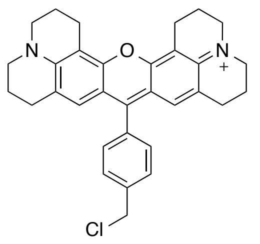

Rho-BH

Figure S3. SILAC-based competitive chemical proteomic method for non-directed target protein ID. (a) Workflow chart of competitive chemical proteomic target identification for non-directed protein target ID for Huh7.5 cells expressing HCV2a replicon. (b) Workflow chart of competitive chemical proteomic target identification for non-directed protein target ID for control Huh7.5 cells.

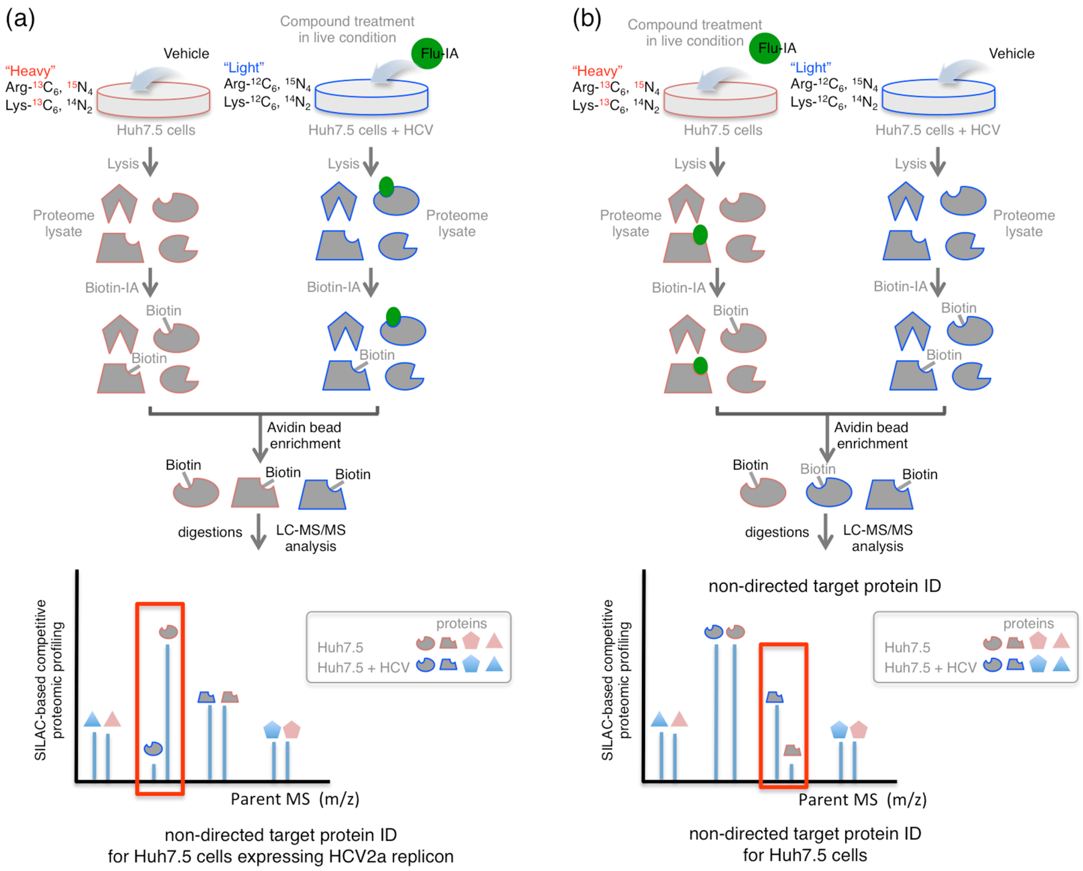

Figure S4. Target protein validation for T-Plastin. Western blot analysis of Huh7.5 cells and Huh7.5 cells expressing HCV2a replicon for T-Plastin and beta-actin. (a) Fluorescence labeling of Flu-IA in Huh7.5 cells without/with expression of HCV2a replicon. Flu-IA was incubated in live Huh7.5 cells with and without expressing HCV2a replicon at 1 μM concentration for 30 min. Total protein 20 μg was loaded on to 12 % SDS-gel, and fluorescence image was obtained using Typhoon fluorescence scanner (excitation: 488 nm laser, emission: 526-SP filter). (b-c) Western blot analysis of T-Plastin. Abundance of T-Plastin was visualized using anti T-Plastin antibody (Abcam: ab45769). β-Actin (Abcam: ab25894) was used as control. (d-e) SILAC-based quantitative analysis of parent MS spectra of T-plastin from non-directed target protein ID; (d) was obtained from Scheme S2-b; (e) was obtained from Scheme S2-a.

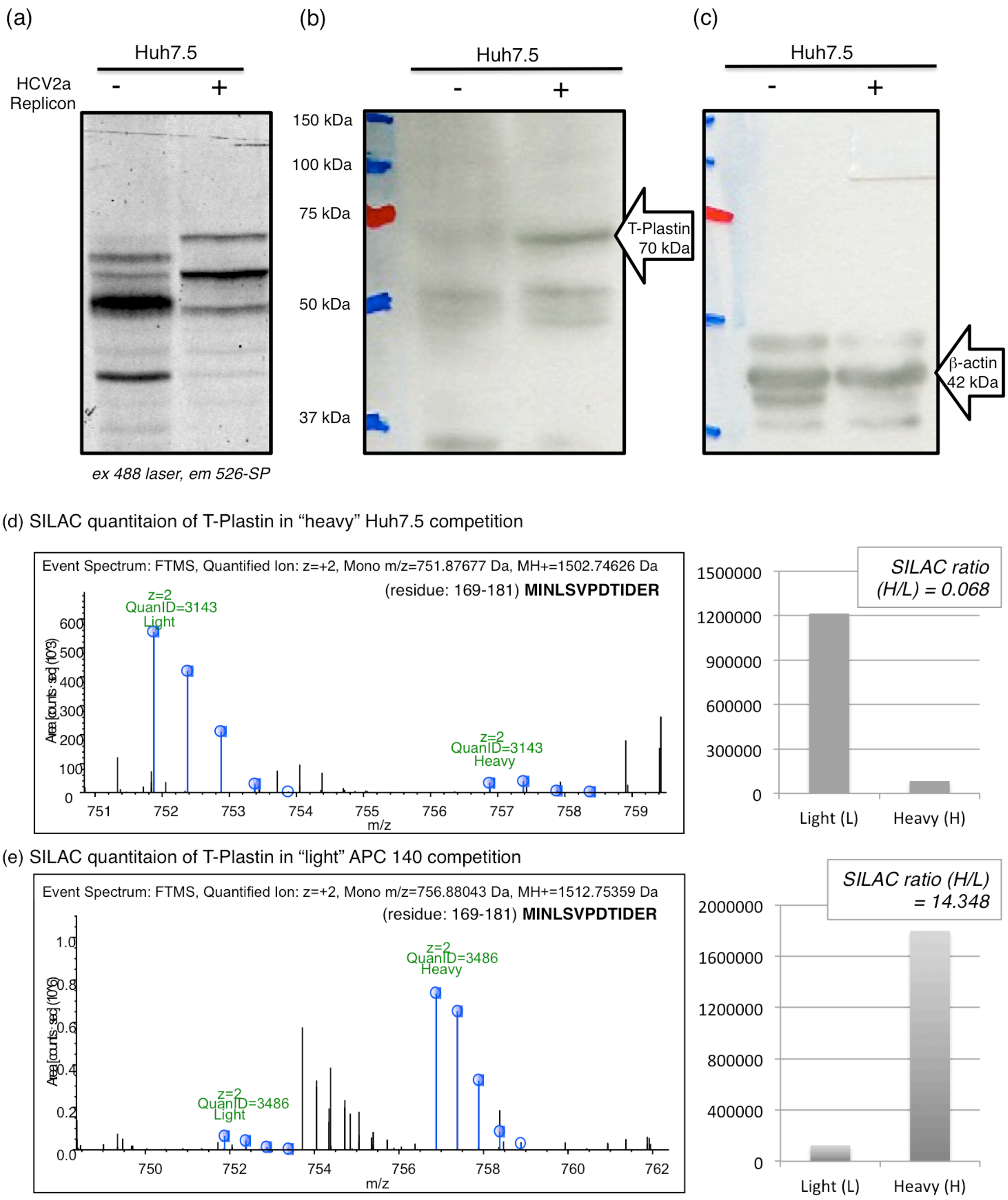

Supplement: Supplementary Information — Supplementary Info [file srep09773-s1.pdf]
